# Data on acylglycerophosphate acyltransferase 4 (AGPAT4) during murine embryogenesis and in embryo-derived cultured primary neurons and glia

**DOI:** 10.1016/j.dib.2015.11.033

**Published:** 2015-11-24

**Authors:** Ryan M. Bradley, Emily B. Mardian, Phillip M. Marvyn, Maryam S. Vasefi, Michael A. Beazely, John G. Mielke, Robin E. Duncan

**Affiliations:** a University of Waterloo, Department of Kinesiology, Faculty of Applied Health Sciences, 200 University Avenue W., BMH 1110, Waterloo, Ontario, Canada N2L 3G1; b University of Waterloo, School of Pharmacy, 10A Victoria Street S., PHR4007, Kitchener, Ontario, Canada N2G 1C5; c University of Waterloo, School of Public Health and Health Systems, Faculty of Applied Health Sciences, 200 University Avenue W., BMH2308, Waterloo, Ontario, Canada N2L 3G1

## Abstract

Whole mouse embryos at three developmental timepoints, embryonic (E) day E10.5, E14.5, and E18.5, were analyzed for *Agpat4* mRNA expression. Primary cortical mouse cultures prepared from E18.5 mouse brains were used for immunohistochemistry. Our data show that *Agpat4* is differentially expressed at three timepoints in murine embryogenesis and is immunodetectable in both neurons and glial cells derived from the developing mouse brain. This paper contains data related to research concurrently published in Bradley et al. (2015) [1].

**Specifications Table**

**Table:** 

Subject area	*Biology*
More specific subject area	*Lipid biochemistry*
Type of data	*Figure, graph*
How data was acquired	*Confocal fluorescence microscopy for AGPAT4 localization, real time PCR (qPCR) and deltaCt analysis for Agpat4 gene expression.*
Data format	*Raw (primary neural culture), analyzed (qPCR)*
Experimental factors	*Mouse embryos were harvested at 3 developmental timepoints*
Experimental features	*Agpat4 expression was determined by qPCR and SEM analysis, AGPAT4 localization was determined by confocal fluorescence microscopy*
Data source location	*University of Waterloo, Waterloo, Ontario, Canada*
Data accessibility	*Data is provided within the article*

**Value of the data**

• These data show that AGPAT4 is variably expressed during murine embryogenesis, suggesting possible importance during fetal development.
• These data show that AGPAT4 is immunodetectable in both cortical neurons and glial cells.
• These data are valuable to researchers interested in investigating phospholipid synthesis during murine embryogenesis.
• These data are valuable to researchers interested in phospholipid synthesis in brain cells.

## 1 Data

**Fig. 1:**
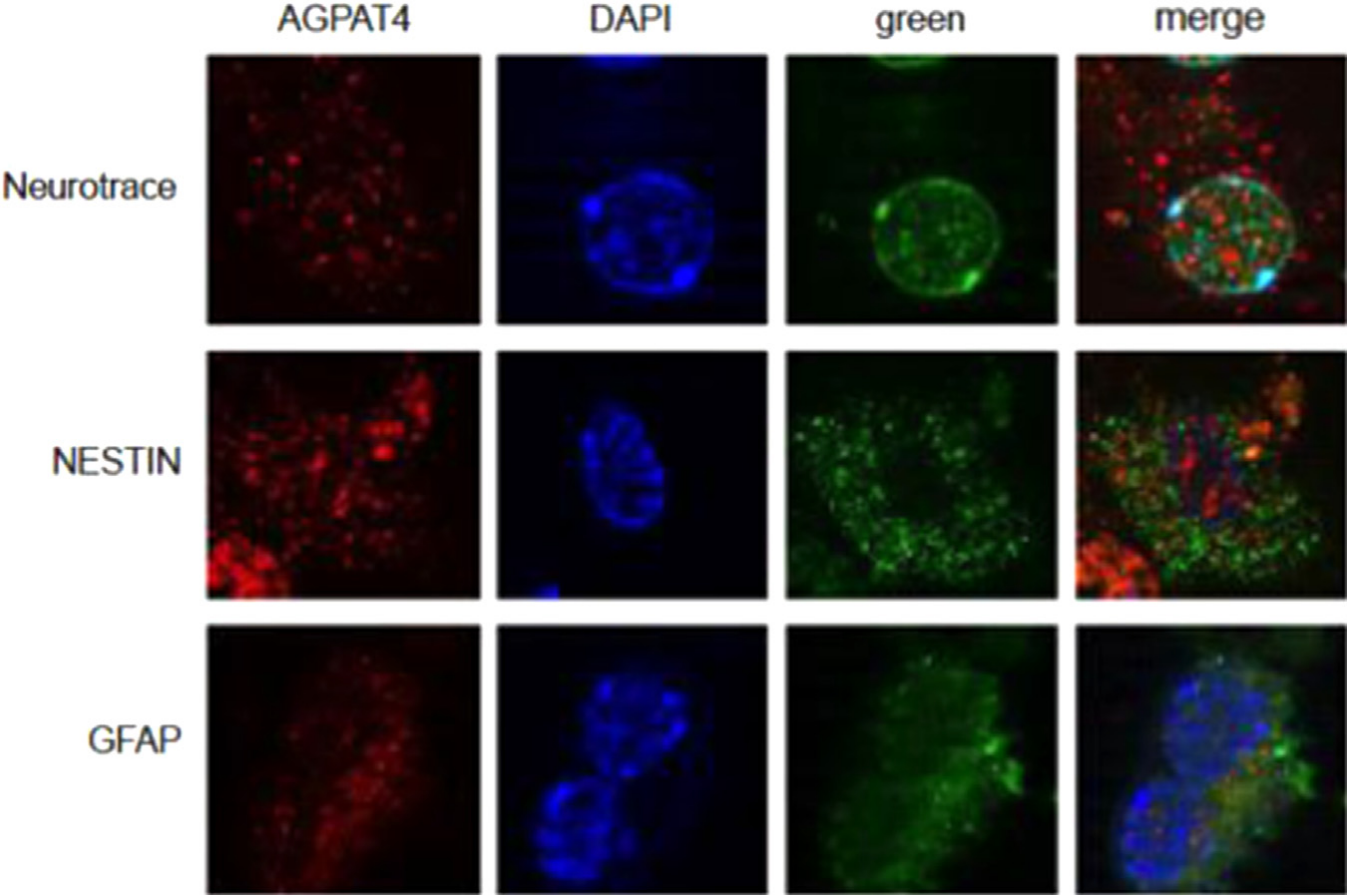
AGPAT4 is detected in both neurons and glial cells**.** Representative images showing immunodetection of AGPAT4 (in red, first column) in a mixed culture of primary cortical neurons and glial cells. Neurons were identified by co-staining with green fluorophore-labeled Nissl stain (Neurotrace®, top row), or by detection of the neuron-specific protein NESTIN (also in green, middle row). Glial cells were identified by detection of the glial marker GFAP (green, bottom row). Nuclei were stained blue with DAPI (second column). Merged wells (last column) show overlap of neuronal or glial cell markers in green with immunoreactive AGPAT4 in red.

Acylglycerophosphate acyltransferase/lysophosphatidic acid acyltransferase (AGPAT/LPAAT) proteins catalyze the second step in the *de novo* synthesis of glycerophospholipids and triacylglycerols [2]. Largely identified through sequence homology, eleven AGPATs have been found in mice and humans [3]. A previous report by our laboratory identified AGPAT4 as a mitochondrial lysophosphatidic acid acyltransferase that is most highly expressed in brain [1]. To determine if AGPAT4 protein is immunodetectable in neurons and/or in glia, we performed immunohistochemistry on mixed cultures of primary cortical cells derived from brains of E18.5 embryonic mice. Cells were grown on coverslips for 7 days in Neurobasal® medium with B27 supplementation to promote neuronal differentiation. Primary neurons were identified as cells expressing the neuron-specific intermediate filament NESTIN, or cells positively co-staining with the fluorescent green Nissl stain Neurotrace 500/525®. Glial cells were identified as cells that were immunopositive for glial fibrillary acidic protein (GFAP). AGPAT4 showed a diffuse, punctate staining (Fig. 1) and was found to co-localize in cells that were identified as positive for either Nissl stain or immunodetectable NESTIN, indicating the presence of this enzyme in primary cortical neurons (Fig. 1). AGPAT4 was also detected in cells that co-express GFAP, indicating that it is also found in glial cells (Fig. 1).

**Fig. 2:**
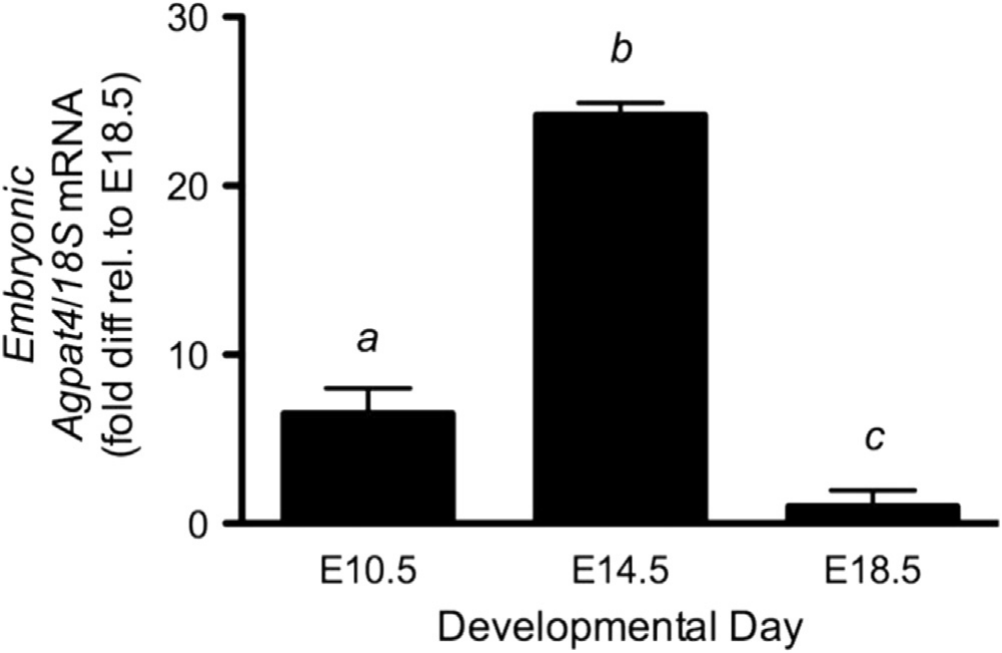
*Agpat4* mRNA is regulated during murine embryogenesis**.***Agpat4* mRNA expression in whole mouse embryos harvested at embryonic (E) developmental day 10.5, 14.5, and 18.5 (*n*=4–6). Data are mean±S.E.M. ^*a vs. b; b vs. c*^*P*<0.001; ^*a vs. c*^*P*<0.05.

Embryogenesis is a time of rapid cellular and organellar growth, which supports organogenesis. Development of cell membranes for expansion of specialized structures such as the central nervous system requires phospholipid biosynthesis, which requires the function of AGPATs. To determine whether *Agpat4* mRNA is regulated during embryogenesis, total RNA was isolated from mouse embryos on developmental days E10.5, E14.5, and E18.5, and analyzed by RT-qPCR for relative *Agpat4* mRNA expression normalized to *18s* expression by the delta Ct method (Fig. 2). *Agpat4* was upregulated 3.7-fold at developmental day E14.5 as compared to day E10.5. *Agpat4* mRNA levels then decreased to only 4% of developmental day 14.5 levels immediately prior to birth, on day E18.5.

## 2 Experimental design, materials and methods

### 2.1 Primary neural culture

Fetal mice at embryonic day E18.5 were decapitated in a cell culture dish on ice containing dissection media (HBSS with 30 mM HEPES, 0.6% w/v glucose, 2% w/v sucrose, final pH 7.4). Brains were dissected out and transferred to a 15 mL conical tube, washed 3× with ice-cold dissection media, and the cortex was separated then incubated with 2 mL of pre-warmed 0.25% trypsin-EDTA for 20 min at 37 °C in a humidified cell culture incubator with 5% CO_2_. Post-incubation, cells were centrifuged at 1000*g* for 5 min, trypsin was removed, and samples were washed once with warm dissection media. Brain samples were then re-suspended by pipetting in 2 mL of warm plating media (DMEM/F12+10% horse serum+10% FBS+1% penicillin–streptomycin) until a homogenous mixture was achieved. The homogenate was strained using a 100 μm nylon cell strainer into a 50 mL conical tube, and centrifuged at 1000*g* for 5 min at 4 °C. The pellet, containing mixed cortical neurons and glial cells, was re-suspended in 2 mL of plating media, and cells were seeded onto glass coverslips pretreated with poly-D-lysine and incubated at 37 °C with 5% CO_2_ for 3 h. Once cells attached to the plate, 50% of plating media was removed and supplemented with feeding media (Neurobasal media+1% B27 supplement) to support the differentiation of primary neurons.

### 2.2 Immunofluorescence

Primary embryonic neurons and glial cells were isolated and grown on coverslips pretreated with poly-D-lysine, then fixed with 4% paraformaldehyde for 10 min, washed with PBS, and permeabilized with 0.5% Triton X-100 for 5 min at room temperature. Cells were then washed with PBS and blocked with 5% goat IgG serum in PBS. After 1 h, blocking serum was removed, and cells were incubated at room temperature for an additional hour with rabbit anti-AGPAT4 antibody (Bioss, Woburn MA), diluted 1:100 in PBS, alone or in combination with mouse anti-GFAP (1:500 dilution) or mouse anti-NESTIN antibodies (1:500 dilution) (Cell Signaling, Danvers MA). Cells were then washed with PBS, and incubated for 1 h at room temperature with Alexa Fluor® 488-conjugated anti-mouse IgG (Cell Signaling, Danvers MA), or stained with Neurotrace® 500/525 Green Fluorescent Nissl Stain (1:100 dilution in PBS) for 20 min at room temperature according to the manufacturer׳s protocol (Life Technologies, Carlsbad CA). Cells were then washed repeatedly with 0.1% Triton X-100 in PBS followed by PBS alone, stained with DAPI (1 μg/mL) for 15 mi, and mounted on glass microscope slides using Prolong Antifade.

### 2.3 RNA extraction, reverse transcription (RT) PCR, and real time (q) PCR

Total RNA was isolated from whole mouse embryos at embryonic (E) days E10.5, E14.5, and E18.5 using TRIzol® Reagent and quantified using a Nanodrop 2000 Spectrophotometer (Thermo Scientific, Waltham MA). cDNA was synthesized from 2 μg of RNA by oligo(dT) priming using SuperScript II Reverse Transcriptase according to the manufacturer׳s protocol. Expression of *Agpat4* mRNA was determined using 1 μL of cDNA with Taqman gene expression assays (Mm00509777_m1 for *Agpat4* and Mm04277571_s1 for *18S*) in a CFX-96 Connect Real Time PCR Detection System (BioRad, Hercules CA). Gene expression levels were normalized to *18S* and quantified by the ΔΔCt method.

### 2.4 Statistical analysis

The data are expressed as mean±S.E.M. Statistically significant differences between two groups were assessed by Student׳s *t* test. Significance is accepted at *P*<0.05.
