# Supplementary material for: Data on acylglycerophosphate acyltransferase 4 (AGPAT4) during murine embryogenesis and in embryo-derived cultured primary neurons and glia
Source: Data Brief. 2015 Nov 24;6:28–32. doi: 10.1016/j.dib.2015.11.033 (PMC4683321; doi:10.1016/j.dib.2015.11.033)
Supplement: Supplementary file 1 — Supplementary material [file mmc1.zip › coi_disclosure_M Beazely COMPLETED.pdf]

Warning: This form is not supported with the current version of Acrobat or Adobe Reader.

Upgrade to the latest version for full support.
